# Characterization of the gut microbiome of Puerto Ricans with Alzheimer’s disease based on their Apolipoprotein E genotype

**DOI:** 10.1002/alz.095590

**Published:** 2025-01-09

**Authors:** Gerianne Olivieri‐Henry, Vanessa Sepulveda, Carlos Alberto Herrero‐Rivera, Cecilia Michelle Soler‐Llompart, Javier A. Ruiz‐Adames, Michel Santiago‐Berríos, Ana Cecilia Sala‐Morales, Hiram Morales, Filipa Godoy‐Vitorino

**Affiliations:** ^1^ University of Puerto Rico, Medical Sciences Campus, School of Medicine, San Juan, PR USA; ^2^ University of Puerto Rico, Río Piedras Campus, San Juan, PR USA

## Abstract

**Background:**

Alzheimer’s disease (AD) is a neurodegenerative condition characterized by a gradual decline in mental function, progressing to death. Evidence suggests the apolipoprotein E (ApoE) gene influences Alzheimer’s risk. Allele E2 is neuroprotective, E3 is neutral, and E4 is associated with a higher genetic risk for AD. Our objective is to study the gut microbiome of Puerto Ricans with AD compared to unimpaired cognitive controls and its association with ApoE allele variants.

**Methods:**

With IRB # 2290033626, we recruited 98 participants, 50 with AD and 48 controls, who underwent clinical and cognitive assessments. Fecal samples were collected for genomic DNA extractions, followed by 16S rRNA genes (V4 region) amplification. ApoE genotyping was done at the PR‐INBRE CRI genomics core, using real‐time PCR TaqMan‐BHQ probes.

**Results:**

Analyses showed no significant differences in bacterial diversity and richness when comparing ApoE genotypes. However, participants with at least one E2 allele showed higher levels of *Firmicutes*, such as *CAG:352* and *NK4A214 group*, while those with at least one E4 allele had higher levels of *Euryarchaeota* and *UBA1819*. When comparing the ApoE genotypes of the AD participants, we found a significant difference in microbial richness (Faith PD pairwise p value = 0.018) between E2E3 and E2E4. Additionally, AD participants with the E2E3 genotype had higher levels of *Fusobacteriota* and *Desulfobacterota* than those with at least one E4 allele. Moreover, we found no significant differences in bacterial diversity and richness when comparing the control participants based on their ApoE genotype. Nevertheless, controls with the E3E4 genotype had higher levels of *Odoribacter* and *Oscillibacter*, while those with E2E4 had a higher density of *Anaerotruncus*, *Desulfovibrio*, and *Faecalibaculum*.

**Conclusion:**

Our study, the first of its kind in Puerto Rico, combines a chronic disease in an aging population with high‐resolution gut microbiome analyses. While overall bacterial diversity and richness did not differ significantly across ApoE genotypes, distinct microbial compositions were observed. This pioneering work in a developing study area may open the possibility for preventive microbiome‐based therapies that could result in a clinical benefit for patients with/without AD.

